# Transcriptomic and proteomic analysis of pyrethroid resistance in the CKR strain of *Aedes aegypti*

**DOI:** 10.1371/journal.pntd.0009871

**Published:** 2021-11-01

**Authors:** Haina Sun, Robert W. Mertz, Letícia B. Smith, Jeffrey G. Scott

**Affiliations:** 1 School of Basic Medicine and Biological Sciences, Soochow University, Suzhou, Jiangsu China; 2 Department of Entomology, Comstock Hall, Cornell University, Ithaca, New York, United States of America; 3 Laboratory of Malaria and Vector Research, NIAID, National Institutes of Health, Rockville, Maryland, United States of America; University of Cincinnati, UNITED STATES

## Abstract

*Aedes aegypti* is an important vector of human viral diseases. This mosquito is distributed globally and thrives in urban environments, making it a serious risk to human health. Pyrethroid insecticides have been the mainstay for control of adult *A*. *aegypti* for decades, but resistance has evolved, making control problematic in some areas. One major mechanism of pyrethroid resistance is detoxification by cytochrome P450 monooxygenases (CYPs), commonly associated with the overexpression of one or more CYPs. Unfortunately, the molecular basis underlying this mechanism remains unknown. We used a combination of RNA-seq and proteomic analysis to evaluate the molecular basis of pyrethroid resistance in the highly resistant CKR strain of *A*. *aegypti*. The CKR strain has the resistance mechanisms from the well-studied Singapore (SP) strain introgressed into the susceptible Rockefeller (ROCK) strain genome. The RNA-seq and proteomics data were complimentary; each offering insights that the other technique did not provide. However, transcriptomic results did not quantitatively mirror results of the proteomics.

There were 10 CYPs which had increased expression of both transcripts and proteins. These CYPs appeared to be largely *trans*-regulated, except for some *CYP*s for which we could not rule out gene duplication. We identified 65 genes and lncRNAs as potentially being responsible for elevating the expression of CYPs in CKR. Resistance was associated with multiple loci on chromosome 1 and at least one locus on chromosome 3. We also identified five CYPs that were overexpressed only as proteins, suggesting that stabilization of CYP proteins could be a mechanism of resistance. Future studies to increase the resolution of the resistance loci, and to examine the candidate genes and lncRNAs identified here will greatly enhance our understanding of CYP-mediated resistance in *A*. *aegypti*.

## Author summary

*Aedes aegypti* is an important vector of human viral diseases and is commonly controlled using pyrethroid insecticides. This has led to the evolution of insecticide resistance in this mosquito via two main mechanisms: increased detoxification of pyrethroids mediated by cytochrome P450 proteins (CYPs) and mutations in the voltage sensitive sodium channel (*Vssc*, gene for the target site of pyrethroids). While much is known about the *Vssc* mutations, the mutation(s) causing the CYP-mediated resistance has been elusive. We used a combined transcriptomic and proteomic approach to try to identify the molecular basis of CYP-mediated resistance in the highly resistant CKR strain. We identified CYPs that were overexpressed in the CKR strain, and for most of these the increased expression was due to a *trans*-acting factor. We identified 65 transcription factors or long non-coding RNAs (lncRNAs) that may play a role in the increased expression of these CYPs. We also found evidence that stabilization of CYP proteins could be a mechanism of resistance.

## Introduction

*Aedes aegypti* vectors four important human disease viruses: dengue, yellow fever, Zika and chikungunya. Given that this mosquito has a wide global distribution, has high vector competence for several arboviruses, frequently bites humans and thrives in urban environments, it poses a serious risk to human health [1]. For example, dengue, the most devastating virus vectored by *A*. *aegypti*, is estimated to be a risk to over 50% of the world’s population [2], and Zika has generated a great deal of human health concern since it arrived in the Americas in 2014 [3–7].

Due to their efficacy and fast action, the use of insecticides has often been the only feasible control strategy for vector-borne diseases. However, insecticide resistance is an increasing problem for vector control [8–10]. Detection and monitoring of resistance is important if we are to mitigate the evolution of insecticide resistance. Identification of the mutations responsible for resistance is a necessary first step for the development of sensitive, high-throughput assays for resistance detection.

Pyrethroid insecticides have been the primary means of controlling adult *A*. *aegypti* populations to suppress arbovirus outbreaks for decades, but resistance to pyrethroid insecticides has become a global problem [8,10–13]. Based on numerous studies, two mechanisms of pyrethroid resistance in *A*. *aegypti* are prevalent: cytochrome P450 (CYP)-mediated detoxification (the causal mutation(s) are not known) and mutations in the target site (*voltage-sensitive sodium channel*, *Vssc*) [8,14–19].

CYP-mediated resistance is commonly found in insects. This resistance mechanism is unequivocally demonstrated by isolation of endoplasmic reticulum (a centrifugal fraction termed microsomes, which contain CYPs) and demonstrating NADPH-dependent (i.e. CYP-mediated) metabolism of the insecticide is higher in the microsomes of the resistant strain [20]. In *A*. *aegypti*, CYP-mediated resistance is associated with increased levels of total cytochrome P450s and/or with increases in expression of one or more CYPs [8]. However, the mutation(s) responsible for this resistance has remained elusive. This is because there are ~160 *CYP*s in *A*. *aegypti* [21] and the factors regulating CYP expression in this species are largely unknown. Our inability to identify the mutations responsible for CYP-mediated resistance constrains our understanding of multiple aspects of this trait (population genetics, fitness costs, etc.).

Another challenge to understanding CYP-mediated resistance is that expression levels are often measured at the transcript level, rather than at the protein level. This has been commonly done, despite the fact that mRNA levels often do not accurately predict the protein levels of that gene [22–35]. Thus, an investigation of the correlation between CYP transcript and protein levels would be highly informative to the field of insect toxicology.

To gain a greater understanding of the molecular basis of CYP-mediated resistance we used the pyrethroid resistant CYP+KDR:ROCK (CKR) strain [14] of *A*. *aegypti*. The CKR strain has the resistance mechanisms from the well-studied Singapore (SP) strain [36] introgressed into the susceptible Rockefeller (ROCK) strain genome. CYP-mediated pyrethroid resistance in SP has been demonstrated by *in vivo* metabolism, *in vitro* metabolism (using microsomes) and by synergism with the CYP inhibitor piperonyl butoxide [36]. Although much is known about the principal mechanisms of pyrethroid resistance in this strain, its underlying molecular basis is still largely unknown (except for *kdr*). The goals of this study were to 1) use transcriptomics (RNA-seq) and proteomics to improve our understanding of the molecular basis of CYP-mediated resistance in *A*. *aegypti*, and 2) to determine if the levels of *CYP* transcripts correlate with levels of CYP proteins across strains. To enhance our chances of success, we compared the CKR, SP and ROCK strains. We found that CYP-mediated resistance in CKR (and SP) is associated with the increased expression level of 10 CYP proteins and we identified a list of candidate genes and long non-coding RNAs (lncRNAs) for future investigation. These candidates include overexpression of groups of CYPs (potentially due to gene amplification), transcription factors and lncRNAs. The lncRNAs were included because of the importance they play in gene regulation [37]. We also identify stabilization of CYP proteins as a potential mechanism of resistance. Future studies to increase the resolution of the resistance loci, and to examine the candidate genes identified here will help move our understanding of CYP-mediated resistance in *A*. *aegypti* forward.

## Materials and methods

### Mosquito strains

Three strains of mosquitoes were used in this study: ROCK is a laboratory susceptible strain [38], SP is a field-collected and laboratory selected permethrin resistant strain [36] and CKR is a permethrin resistant strain that is congenic to ROCK, but has *knockdown resistance* (*kdr*) (S989P + V1016G mutations in *Vssc*) plus CYP-mediated resistance mechanisms from the SP strain [39]. We confirmed that the strains had the previously reported levels of resistance (permethrin resistance ratios were 360 and 110 for SP and CKR, respectively) [14,15] prior to starting our experiments. Mosquitoes were reared in a room with temperatures ranging from 25–30°C (median and average = 28), 3–37% (median = 14, average = 16) relative humidity, and a 14L:10D photoperiod. All three strains were reared in parallel for each of the biological replicates for the RNA-seq libraries and microsome preparations (proteomics). About 400 first instar larvae were transferred into a plastic container (29.5 x 23 x 8.4 cm) (Lock&Lock Co., Ltd., Seoul, Korea) in which ~ 1/3 of the lid was cut out and covered with nylon tulle. Each container had 2 L distilled water and larvae were fed with Cichlid Goldfish food pellets (Hikari, Hayward, CA, USA) as needed. Adults were released in cages (35 x 25 x 25 cm) and provided with 10% sugar water. For colony maintenance, female mosquitos were blood fed using membrane-covered water-jacketed glass feeders containing cow blood from a local butcher (Owasco Meat Co., Moravia, NY, USA).

### Rationale

In order to understand the molecular basis of CYP-mediated resistance we considered six testable hypotheses (Table 1). One hypothesis (#1) was that resistance was due to a polymorphism in a CYP resulting in an enzyme with a higher rate of detoxification of pyrethroids (this was previously shown for *Cyp6a2* and metabolism of DDT [40] and for *CYP9A186* and abamectin metabolism [41]). Two hypotheses involved increased expression of one CYP due to a mutation in the CYP “promoter” (hypothesis #2, as found for *CYP6D1* and metabolism of pyrethroids, [42–44]) or due to gene duplication (hypothesis #3, as found for resistance to neonicotinoids due to duplication of *CYP6CY3* [45]). Two hypotheses involved increased expression of multiple CYPs due to a mutation in a “switch” (i.e. transcription factor or lncRNA, hypothesis #4) or a mutation in the “promoter” of a “switch” (hypothesis #5). We also hypothesize (#6) that resistance could be due to stabilization of one or more CYP proteins, as this has been found as a mechanism of CYP overexpression due to disruption of P450 proteolytic turnover via endoplasmic reticulum-associated degradation (ERAD) pathway in mammals [46–48]. It would also be possible that resistance could be due to stabilization of a *CYP* transcript although this would be impossible to distinguish from hypotheses #3, 4 or 5 with our RNA-seq and proteomics data. A schematic of the approaches taken in this study is shown in S1 Fig.

**Table 1:** Six hypotheses to explain increased CYP-mediated resistance that are testable with RNA-seq and/or proteomic data.

Hypothesis	Evolutionary Event	Resulting change in biological function leading to resistance	Predicted observation(s) in transcriptomic & proteomic data
1	Non-synonymous polymorphism (SNP or in-frame indel) in a CYP	CYP has increased detoxification	Resistance associated^*a*^ SNP in a CYP, but no associated change in transcript or protein level)
2^*b*^	Mutation (SNP or indel) in the promoter of a CYP	Increase in expression of that CYP	Increased expression of a CYP (transcript and protein)
3^*b*^	Gene duplication	Increase in expression of the duplicated CYPs ^*c*^	Increased expression of the CYP(s) (transcripts and proteins)
4	Non-synonymous polymorphism (SNP or indel) in switch^*d*^	Switch^*d*^ has different regulatory properties or becomes a null, leading to increased CYP expression	Altered expression of the genes controlled by the switch including increased expression of some CYPs (transcripts and protein)
5	Mutation (SNP or indel) in the promoter of a switch	Change in amount of the switch, resulting in increased expression of one or more CYPs^*e*^	Altered expression of the switch, and of the genes controlled by the switch, including increased expression of some CYPs (transcripts and proteins)
6	Decreased degradation rate of CYP(s)	One or more CYPs are stabilized, leading to increased expression	Increased expression of one or more CYP proteins, but no increase in the expression of their transcripts

^*a*^Polymorphism found as homozygous in SP and CKR, but not found in the insecticide susceptible ROCK or Liverpool strains.

^*b*^Hypotheses 2 and 3 would result in similar patterns in the RNA-seq and proteomics data sets, but would potentially be differently resolved in our mapping experiments.

^*c*^This could be a single *CYP* or a group of tightly clustered CYPs.

^*d*^This could be a transcription factor or a lncRNA.

^*e*^The number of genes affected is unknown, but presumably would be everything that the switch controls.

### RNA extraction and cDNA library preparation

There were four biological replicates for each strain. For each biological replicate, the eggs of each strain were hatched once a week for a four-week period (one replicate done each week), and larvae were reared under the laboratory conditions described above. Adult *A*. *aegypti* 4–7 days old were anesthetized by chilling to 4°C and then kept on an ice-cold surface. Total RNA was extracted from 20 female (mated, but not blood fed) abdomens that were separated using forceps and the intact abdomens were immediately placed in 0.5 mL TRIzol (Invitrogen, Carlsbad, CA, USA). Abdomens were used because they were the body region used to make microsomes and demonstrate increased CYP-mediated resistance in the SP strain [36]. Total RNA was extracted as previously described [14]. RNA was quantified with a NanoDrop 2000 spectrophotometer (Thermo Fisher, San Jose, CA, USA) and a Qubit Fluorometer (Invitrogen). Strand-specific RNA-seq libraries were prepared for sequencing by Polar Genomics (Ithaca, NY, USA).

### Differentially expressed gene analysis with RNA-seq data

Sequencing was performed on an Illumina NextSeq 500 sequencer in one lane of 75-bp single-end-read runs in the Biotechnology Resource Center of Cornell University and the sequences were generated by Illumina pipeline software v2.18 in a fastq format. The raw data were quality checked using FastQC (version 0.11.5, https://www.bioinformatics.babraham.ac.uk/projects/fastqc/). TopHat (v2.1.1, [49]) was used to align the Illumina reads against the *A*. *aegypti* [susceptible Liverpool (LVP) strain] genome (version AaegL5.1) with Bowtie2 (v2.2.8.0) [50], allowing a maximum of two mismatches. The edgeR package [51] was used for multidimensional scaling (MDS) plot and analysis of differential gene expression of the RNA-seq data using the edgeR_count.xls file generated from mapping [51,52]. The gene expression profile was analyzed with edgeR’s generalized linear models. Only genes with a count per million (CPM) value greater than 1 in at least 4 samples were used for the relative expression levels between two strains. To obtain a list with a feasible number, the genes were counted as differentially expressed if the log_2_ fold-change (FC) was ≥ 1 and had a false discovery rate (FDR) ≤ 0.01. Volcano plots were generated with the log_2_ (FC) value and -log_10_ (FDR) for three comparisons between two different strains using R [53] software.

### Single nucleotide polymorphism (SNP) analysis with RNA-seq data

Single nucleotide polymorphism (SNP) analysis was carried out using the transcriptomic data with the LVP genome (AaegL5.1) as the reference. The Sentieon Genomic Tool (v201704.01) [54] was used for variant calling from the merged bam file of four biological replicates of each strain. The variants with phred-scaled confidence lower than 30 (QUAL score < 30) were filtered out. The single nucleotide polymorphisms (SNPs) and indels were separated using SelectVariants tool in GATK (v4.0.1.1). The software snpEff (4.3t) was used to predict the effects of SNPs and indels on genes [55]. To search for resistance loci present in both the SP and CKR strains, we filtered resistance associated homozygous SNPs (in both SP and CKR) (using 10 Mb windows) that were different from both ROCK and the genomic reference LVP strains.

### Isolation of microsomes

Microsomes (centrifugation fraction containing smooth and rough endoplasmic reticulum) from the SP strain were found to metabolize permethrin at a more rapid rate than a susceptible strain [36]. Thus, microsomes were used as our source for proteomic analysis. In addition, microsomes provide a feasible number of proteins for a proteomic study [56,57]. Microsomes were isolated using a protocol originally developed for house flies [58] and adapted for mosquitoes [59]. Female abdomens were collected as described above and at the same time (i.e. same mosquito cohort used for both RNA extraction and microsome preparation). Intact abdomens were immediately placed in ice cold homogenization buffer (0.1 M sodium phosphate pH 7.5, 10% glycerol (v/v), 1 mM EDTA, 1 mM PMSF, 1 mM PTU, and 0.1 mM DTT). Approximately 1200 abdomens were collected for each replicate of SP and CKR, and 1400 abdomens were used for each replicate of ROCK (anticipating a lower total CYP content). Abdomens were homogenized in 10 mL of homogenization buffer using a 15 mL glass/Teflon homogenizer until all abdomens were disrupted. The homogenate was filtered through cheesecloth into a chilled centrifuge tube, then the solid material was added back to the homogenizer with 10 mL fresh homogenization buffer. After 5–10 additional passes with the pestle the homogenate was filtered through cheesecloth into the same centrifuge tube. The homogenizer was then rinsed with 5 mL homogenization buffer and filtered through cheesecloth into the centrifuge tube. The filtered homogenate was centrifuged at 10,000 g at 4°C for 20 minutes. The supernatant was filtered through cheesecloth and then centrifuged at 100,000 g at 4°C for 1 hour. The supernatant was discarded and the pellet transferred to a chilled 1 mL glass/Teflon homogenizer with 0.5 mL resuspension buffer (0.1 M sodium phosphate pH 7.5, 20% glycerol (v/v), 1 mM EDTA, 1 mM PMSF, and 0.1 mM DTT). This was homogenized with 5–10 passes or until fully resuspended and transferred to a cryovial. Another 0.5 mL resuspension buffer was added to the homogenizer and passed 5–10 times to resuspend and rinse out any remaining material, then this was added and mixed into the cryovial. The protein content of the microsomes was quantified using the Bio-Rad Protein Assay Dye Reagent (Hercules, CA) as per the manufacturer’s instructions and the microsomes were stored at -70°C.

### Cytochrome P450 and cytochrome *b*_5_ assays

Microsomes were assayed for total cytochrome P450s and cytochrome *b*_5_ [60] using a Molecular Devices SpectraMax Plus 384 (San Jose, CA) with a 1 cm path length quartz cuvette and spectra were collected from 400–500 nm. All cytochrome P450 and *b*5 assays were performed in triplicate for each biological replicate. Statistical analyses were carried out with ANOVA followed by a TukeyHSD test using R [61]. Cytochrome b_5_ is important in CYP mediated metabolism because it can act as the second electron donor or may be a required co-factor [43,62].

### Construction of a strain-specific variation informed proteomic database

To maximize peptide detection and gain a higher proteome coverage, a strain-specific protein database was established for ROCK and SP starting from the proteins predicted from the LVP genome (AaegL5.1) and then substituting the variants (strain-specific sequence differences) that were identified in the transcriptomic analysis. The bam files with mapped reads generated from the TopHat aligner for each RNA-seq library were merged for each strain, and variants were called for both strains using samtools (v1.8, http://www.htslib.org/) and bcftools (v1.8, http://samtools.github.io/bcftools/bcftools.html). Alternate fasta sequences for each strain were generated by FastaAlternateReferenceMaker tool in GATK (v4.0.1.1), which replaced the reference bases with the variants supplied by a variant call format (VCF) file. Protein fasta files were generated from the strain-specific transcriptomes using gffread (v0.9.12). The longest isoform of each protein sequence was extracted from the reference genome LVP, and ROCK- or SP-specific protein fasta files. Ultimately, LVP-, ROCK- and SP-specific protein databases containing the longest isoform of each protein were established for protein database searches.

### Protein extraction, digestion and TMT 10plex labeling

Microsomes from all four ROCK replicates and three each of the SP and CKR replicates were submitted to the Biotechnology Resource Center at Cornell University for quantitative analysis with isobaric labels. Microsomes were denatured in a final concentration of 0.1 M phosphate buffer pH 7.4, 8 M urea, and 0.15% SDS. The protein concentration for each sample was determined by Bradford assay [63] using BSA as the standard, and further quantified by running on a precast NOVEX 10% Bis-Tris mini-gel (Invitrogen) along with a series of *E*. *coli* lysates (2, 5, 10, 20 μg/lane). The SDS gel was visualized with colloidal Coomassie blue stain (Invitrogen), imaged with a Typhoon 9400 scanner and ImageQuant Software version TL 8.1 (GE Healthcare, Chicago IL).

Further processing of the proteins was then performed according to Thermo Fisher Scientific’s TMT Mass Tagging Kits and Reagents protocol with a slight modification [64,65]. A total of 50 μg protein of each sample was reduced with 20 mM tris (2-carboxyethyl) phosphine for 1 h at room temperature, alkylated with 20 mM iodoacetamide for 1 h in the dark and then quenched by addition of 20 mM dithiothreitol (DTT). The alkylated proteins were precipitated by adding 6 volumes of ice-cold acetone and incubating at −20°C overnight, and reconstituted in 50 μL of 100 mM triethylammoniumbicarbonate. Each sample was digested with 2.5 μg trypsin for 18 h at 35°C. The TMT 10plex labels (dried powder) were reconstituted with 25 μL of anhydrous acetonitrile (ACN) prior to labeling and added at a 1:2 ratio to each of the tryptic digest samples for labeling for 1 hour at room temperature. The peptides from the 10 samples (4 ROCK samples as controls, 3 SP samples and 3 CKR samples) were mixed with each tag as follows: ROCK (126, 127C, 127N, and 128C), SP (128N, 129C, and 129N), and CKR (130C, 130N, and 131). After label incorporation was checked using an Orbitrap Fusion (Thermo Fisher) by mixing 1 μL aliquots from each sample and desalting with SCX ziptip (Millipore, Billerica, MA), the 10 digested samples were pooled together. The pooled peptides were evaporated to 200 μL and subjected to cleanup by solid phase extraction (SPE) on Sep-Pak Cartridges (Waters, Milford, MA). The eluted tryptic peptides were evaporated to dryness, and ready for the first liquid chromatography (LC) fractionation via high pH reverse phase chromatography as described below.

### High pH reverse phase (hpRP) fractionation

The hpRP chromatography was carried out using a Dionex UltiMate 3000 HPLC system with the built-in micro fraction collection option in its autosampler and UV detection (Thermo Fisher) as reported previously [65–67]. Specifically, the TMT 10plex tagged tryptic peptides were reconstituted in 20 mM ammonium formate pH 9.5, and loaded onto an XTerra MS C18 column (3.5 μm, 2.1 x 150 mm) from Waters, (Milford, MA) with 20 mM ammonium formate pH 9.5 as buffer A and 80% ACN/20% 20 mM ammonium formate as buffer B. The LC was performed using a gradient from 10–45% of buffer B over 30 minutes at a flow rate of 200 μL/min. Forty-eight fractions were collected at 1 minute intervals and pooled into a total of 10 fractions based on the UV absorbance at 214 nm and with multiple fraction concatenation strategy [67]. Each of the 10 fractions was dried and reconstituted in 125 μL of 2% ACN/0.5% formic acid (FA) for nanoLC-MS/MS analysis.

### Nano-scale reverse phase chromatography and tandem mass spectrometry (nanoLC-MS/MS)

The nanoLC-MS/MS analysis was carried out using an Orbitrap Fusion (Thermo Fisher) mass spectrometer equipped with a nanospray Flex Ion Source using high energy collision dissociation (HCD) similar to previous reports [64–66] and coupled with the UltiMate3000 RSLCnano (Dionex, Sunnyvale, CA, USA). Each reconstituted fraction (3 μL for global proteomics fractions) was injected onto a PepMap C-18 RP nano trap column (3 μm, 100 μm × 20 mm, Dionex) at 15 μL/min flow rate for on-line desalting, and separated on a PepMap C-18 RP nano column (2 μm, 75 μm x 25 cm). The labeled peptides were eluted with a 120 min gradient of 4% to 35% ACN in 0.1% FA at 300 nL/min, followed by an 8-min ramping to 95% ACN/0.1% FA and a 9-min hold at 95% ACN/0.1% FA. The column was re-equilibrated with 2% ACN/0.1% FA for 25 min prior to the next run. The Orbitrap Fusion was operated in positive ion mode with nanospray voltage set at 2.1 kV and source temperature at 275°C. External calibration for mass analyzers was performed. For global proteomics fractions, the instrument was operated in data-dependent acquisition (DDA) mode using FT mass analyzer for one survey MS scan for selecting precursor ions followed by 3 second “Top Speed” data-dependent HCD-MS/MS scans for precursor peptides with 2–8 charged ions above a threshold ion count of 20,000 with normalized collision energy of 40%. MS survey scans at a resolving power of 120,000 (fwhm at *m*/*z* 200), for the mass range of m/z 400–1600 with AGC = 4 x 10^5^ and Max IT = 50 ms, and MS/MS scans at 60,000 resolution with AGC = 1 x 10^5^, Max IT = 86 ms and with Q isolation window (m/z) at 1.6 for the mass range m/z 105–2000. Dynamic exclusion parameters were set at 1 within 50 s exclusion duration with ± 10 ppm exclusion mass width. All data are acquired under Xcalibur 3.0 operation software and Orbitrap Fusion Tune 3.0 (Thermo Fisher).

### Data processing, protein identification and data analysis

All MS and MS/MS raw spectra from each set of TMT10-plex experiments were processed and searched using the Sequest HT search engine within Proteome Discoverer 2.2 or 2.3 (PD2.2/3, Thermo Fisher). The *A*. *aegypti* NCBI protein database containing 35,399 entries downloaded on August 30, 2018 was used for an initial database search in PD2.2, revealing multiple alleles of some genes and four CYPs incorrectly annotated in the AaegL5.1 genome (these CYPs were identified from the *Cytochrome P450 homepage* [68]). The ROCK variant-informed protein database consisting of the longest transcript from each gene in AaegL5.1 was found to identify more proteins than a similar unmodified LVP database or SP-specific database. This ROCK-specific protein database, with the incorrectly annotated CYP sequences added, was used for the final database search using PD2.3. The default search settings used for TMT 10plex quantitative processing and protein identification were: two missed cleavages for full trypsin with fixed carbamidomethyl modification of cysteine, fixed TMT 10plex modifications on lysine and N-terminal amines and variable modifications of methionine oxidation, deamidation on asparagine/glutamine residues and protein N-terminal acetylation. The peptide mass tolerance and fragment mass tolerance values were 10 ppm and 0.02 Da, respectively. Identified peptides were filtered for maximum 1% FDR using the Percolator algorithm in PD 2.3 along with additional peptide confidence set to high. The TMT 10plex quantification method within PD2.3 software was used to calculate the reporter ion abundances that were corrected for isotopic impurities. Both razor (assigned to the protein with the largest number of peptide matches) and unique peptides were used for quantitation. Signal-to-noise (S/N) values of peptides, which were summed from the S/N values of the peptide spectrum matches (PSMs), were summed to represent the abundance of the proteins. For relative ratios between two groups, normalization on total peptide amount for each sample was applied. The search result including ratio, p-value, and peptide abundance for each sample was outputted to Microsoft Excel software for further data analysis. The mass spectrometry proteomics data have been deposited to the ProteomeXchange Consortium via the PRIDE [69] partner repository with the dataset identifier PXD025994.

### Correlation of transcript levels vs. protein levels

Correlation of transcript abundance and protein levels were displayed graphically using ggplot2 in R [70]. The correlation coefficients were calculated using the Pearson method using R. The linear model function was used to create the regression line for correlation analysis and the statistics (such as *r*^*2*^ and equation) was summarized using R.

## Results

### Transcriptomic analysis

The RNA-seq libraries generated a total of 428,778,534 reads (S1 Table). Each library ranged from 24–42 million reads. Overall, 89.4% to 90.4% of the total reads from each library mapped to the reference genome at least once. Among those reads that mapped to the genome, 92.1%-92.7% mapped uniquely. A multidimensional scaling (MDS) plot was used to evaluate the level of similarity between different strains and biological replicates. Based on the MDS plot, ROCK and SP were separated along dimension 1 (the x axis), and CKR was in between ROCK and SP in dimension 1, but was separated from ROCK and SP in dimension 2 (the y axis) (S2 Fig), indicating that the strain differences were greater than the batch effects (i.e. replicates) among our libraries. There were >15,500 genes detected in each strain (S2 Table).

### Differentially expressed genes and lncRNAs between susceptible and resistant strains

The differentially expressed genes and lncRNAs were determined in pairwise comparisons using the edgeR package in R [51,52]. A total of 15,541, 15,733 and 15,712 genes and lncRNAs were detected at least once in ROCK, SP and CKR, respectively (S2 Table). After removal of genes with no counts in one strain and the genes with CPM<1 in at least 4 libraries in two strains, 10,595, 10,502 and 10,504 genes and lncRNAs remained and were used to determine relative pairwise expression levels between the SP/ROCK, CKR/ROCK and SP/CKR strains, respectively (S3 Table).

**Fig 1:**
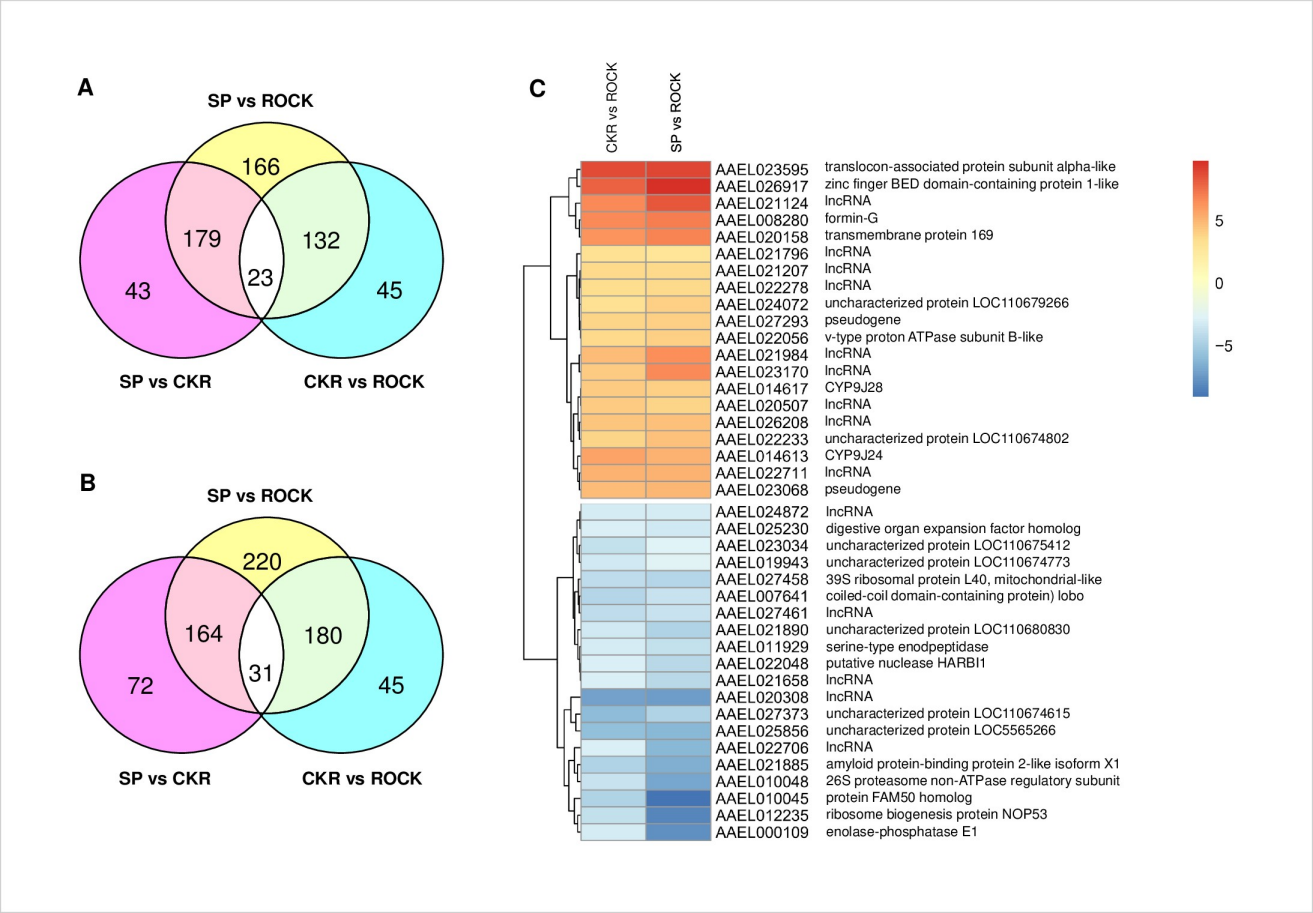
Genes and lncRNAs that were up- or down-regulated between two strains from the RNA-seq experiments. A: Venn diagram of the number of genes and lncRNAs that are significantly up-regulated (log_2_ (FC) ≥ 1 & FDR ≤ 0.01) between two strains; B: Venn diagram of the number of genes and lncRNAs that are down-regulated (log_2_ (FC) ≤ -1 & FDR ≤ 0.01) between two strains; C: Heat map of the top 20 most differentially expressed genes (based on CKR vs. ROCK comparison, but all genes shown were also differentially expressed in SP relative to ROCK). Heat maps were generated using PHEATMAP (https://www.rdocumentation.org/packages/pheatmap/versions/1.0.12/topics/pheatmap).

The numbers of genes and lncRNAs that are differentially expressed between strains are shown in Fig 1. Compared to the susceptible ROCK strain, a total of 500 and 200 genes plus lncRNAs were significantly up-regulated (log_2_ (FC) ≥ 1 and FDR ≤ 0.01) in the SP and CKR strains, respectively (Fig 1A and 1C). For the down-regulated genes and lncRNAs, a total of 595 and 256 were found significantly down-regulated (log_2_ (FC) ≤ -1 and FDR ≤ 0.01) in SP and CKR strains relative to the susceptible ROCK strain, respectively (Fig 1B and 1C). Comparing the resistant strains to ROCK, the number of up-regulated and down-regulated genes and lncRNAs were similar but there were slightly more down-regulated genes plus lncRNAs in both resistant strains.

The resistance mechanisms in CKR were inherited from SP, thus the genes plus lncRNAs found differentially expressed in both resistant strains relative to ROCK were of high interest. There were 155 genes plus lncRNAs that were significantly upregulated in both CKR and SP, relative to ROCK (S4 Table). The most commonly identified were lncRNAs (39), uncharacterized proteins (22), CYPs (14) and putative transcription factors (14). The gene on this list that was most differentially expressed based on the CKR/ROCK log_2_ (FC) was *translocon-associated protein (TRAP) subunit alpha-like* (a single-spanning membrane protein of the endoplasmic reticulum found in proximity of nascent polypeptide chains translocating across the membrane [71]). According to UniProt, “TRAP proteins are part of a complex whose function is to bind calcium to the ER membrane and thereby regulate the retention of ER resident proteins” https://www.uniprot.org/uniprot/P43307. The CYP that was most up-regulated based on the CKR/ROCK log_2_ (FC) was *CYP9J28* (19-fold higher expression in CKR vs. ROCK) which has been shown to be capable of metabolizing permethrin [72]. Based on the genome annotation file (AaegL5.1), 58, 30 and 55 of the up-regulated genes were on chromosome 1, 2 and 3, respectively (S4 Table). It is worth noting that a cluster of 6 up-regulated CYPs from the CYP9 subfamily were located on chromosome 3 (*CYP9J10*, *17*, *24*, *26–28*). In addition, an up-regulated transcription factor AAEL006932 (conserved domain TF_AP-2: pfam03299) is located close to the 5’ end of this CYP cluster. There were 211 genes and lncRNAs that were significantly down-regulated in both CKR and SP, relative to ROCK (S5 Table). The most commonly identified were uncharacterized proteins (49) and lncRNAs (33). There were 13 putative transcription factors and also five CYPs that were down-regulated in the resistant strains (S5 Table). The greatest reduction in expression based on the CKR/ROCK log_2_ (FC) was a lncRNA (AAEL020308). AAEL025856 was also greatly reduced in both resistant strains, and this gene codes for a protein with four RNA recognition motifs (RRMs) which are known to be involved in post-transcriptional gene expression processes including mRNA and rRNA processing, RNA export, and RNA stability [73]. There were 83, 40 and 72 down-regulated genes on chromosomes 1, 2 and 3, respectively (S5 Table). About 8% of the up- or down-regulated genes in both SP and CKR strains were distributed among different contigs that were not assembled into a chromosome. The number of genes with differential expression between chromosomes was not consistent with the size of the chromosomes (i.e. 2>3>1, [74]), suggesting the distribution was not random. Volcano plots showing the significantly up- or down-regulated genes for the three pairwise comparisons between strains are shown in S3 Fig.

### Single nucleotide polymorphism (SNP) analysis

**Fig 2:**
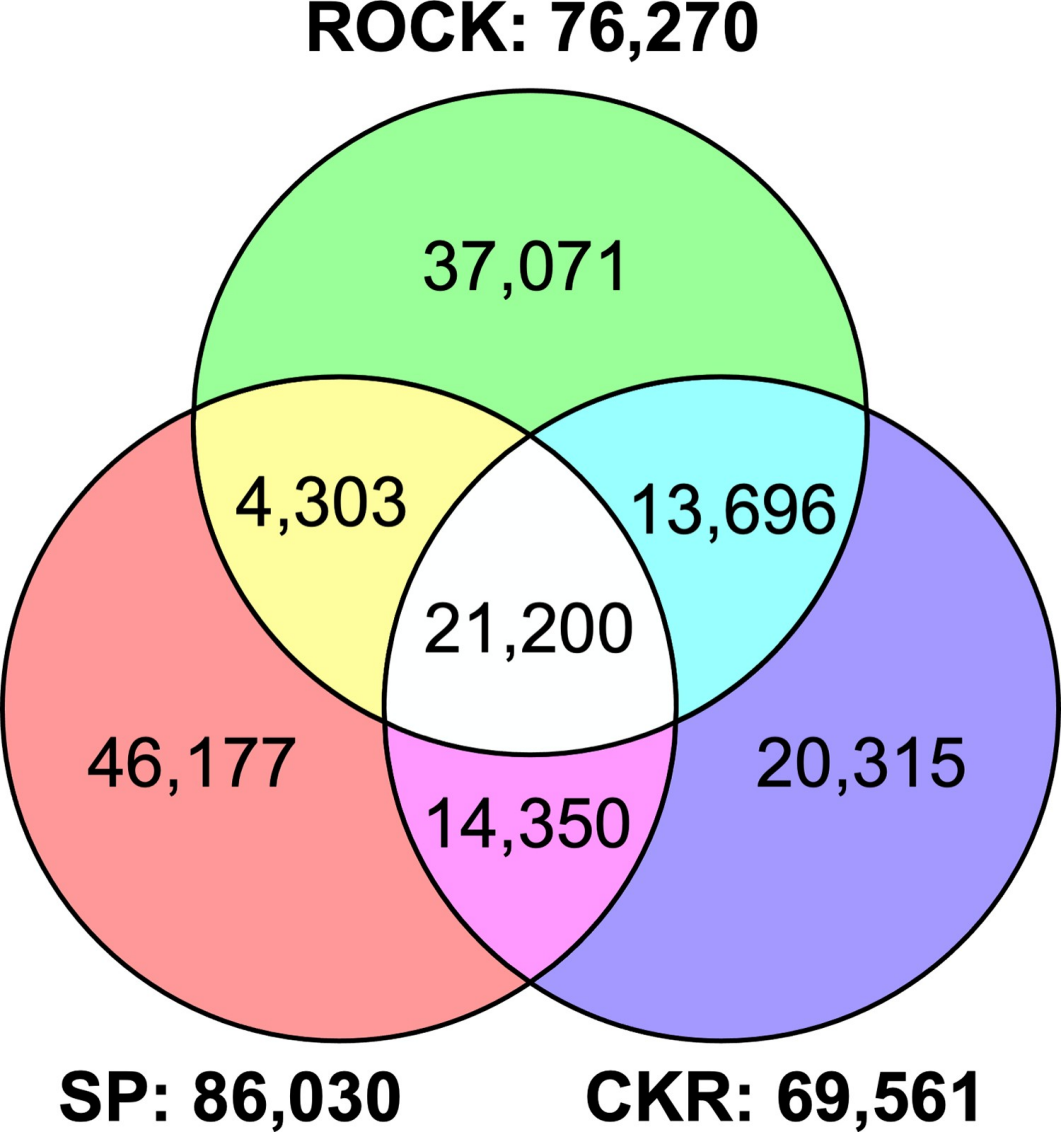
Venn diagram of homozygous single nucleotide polymorphisms (SNPs) called from transcriptomic data in ROCK, SP and CKR strains, relative to the susceptible Liverpool strain.

**Fig 3:**
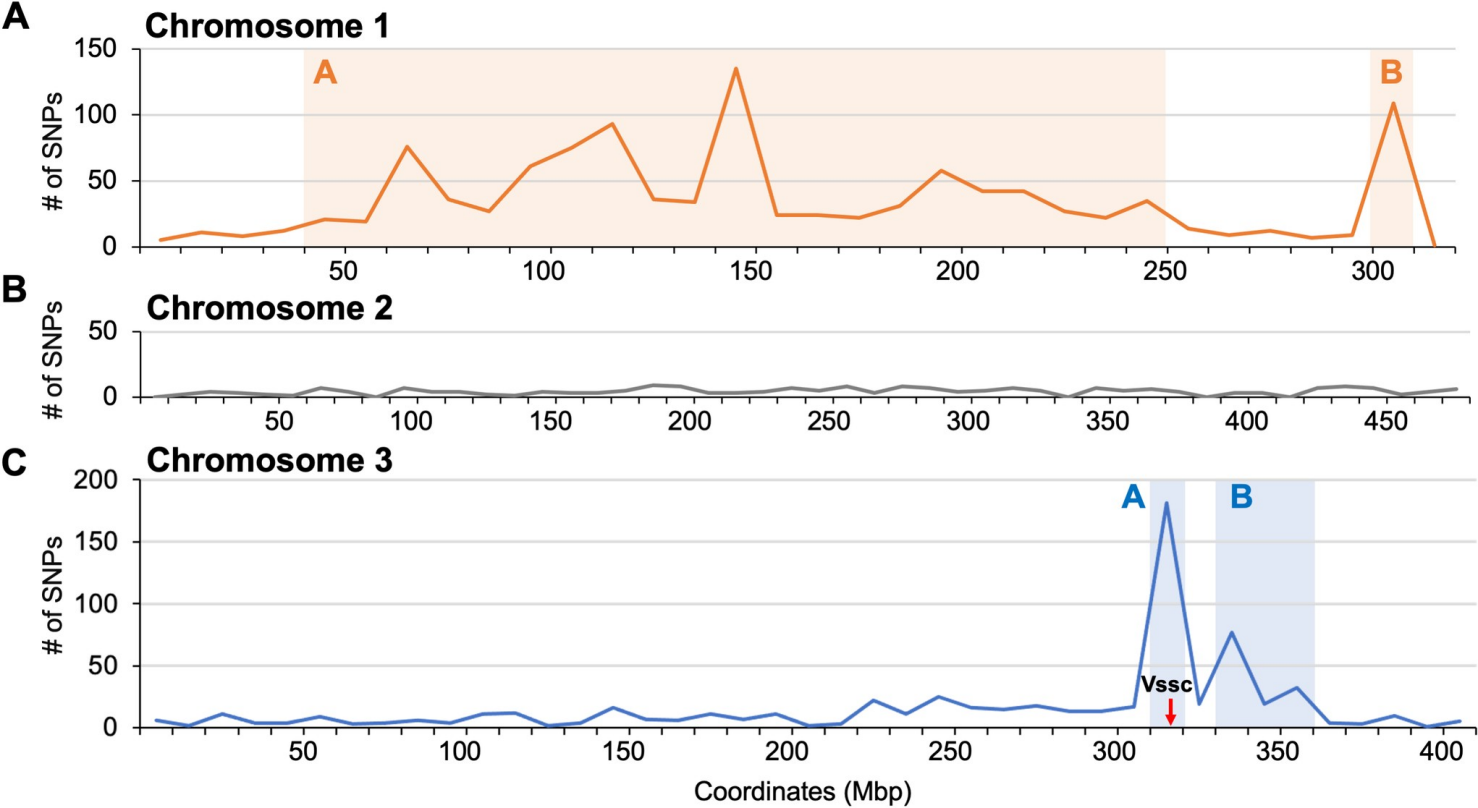
The distribution of potentially resistance associated SNPs across three chromosomes within a 10 Mb window size. The two resistance loci detected on chromosome 1 and chromosome 3 are shown by the orange and blue shaded areas, respectively. Red arrow indicates the coordinate of *Vssc* on chromosome 3.

Compared to LVP, a total of 76,270, 86,030 and 69,561 homozygous SNPs were observed from the transcriptomic data in the ROCK, SP and CKR strains, respectively (Fig 2). In general, the SP strain had more SNPs compared to the LVP strain than ROCK and CKR strains. There were 21,200 homozygous SNPs found to be shared in ROCK, SP and CKR that were different than LVP. We further analyzed the 86,030 SNPs found in SP (relative to LVP) to determine those shared by SP and CKR that were different from ROCK, and identified 2,021 SNPs matching this resistance-related pattern (S6 Table). Based on the chromosomal coordinates of those 2,021 SNPs, 1,137 are on chromosome 1, 204 are on chromosome 2, and 646 are on chromosome 3 (S6 Table). Theoretically, all of these SNPs in CKR were inherited from the SP strain, and potentially show some genetic linkage to resistance. The numbers of resistance-associated SNPs across the three chromosomes (within 10 Mb windows) are shown in Fig 3. As expected, there was a major peak on chromosome 3 with 181 homozygous SNPs in the region of 310 Mb-320 Mb containing the *Vssc* gene (315.9 Mb-316.4 Mb), and the majority (114/181) of the homozygous SNPs were found within 1 Mb region (315 MB-316 Mb) of *Vssc* (Fig 3C). On chromosome 1, there were multiple peaks and two regions having more than 100 homozygous SNPs/10 Mb, suggesting there are multiple resistance loci on chromosome 1 (Fig 3A). In contrast, the 204 homozygous SNPs on chromosome 2 were evenly distributed (Fig 3B), suggesting there were no resistance loci on this chromosome. Overall, the resistance related SNPs we detected suggest multiple resistance loci on chromosome 1, no resistance loci on chromosome 2 and one or two loci on chromosome 3 (one that contained *Vssc*).

The 2,021 homozygous SNPs found in both SP and CKR strains (relative to ROCK and LVP) were annotated with snpEff, and the predicted effects of those SNPs (e.g. synonymous, non-synonymous, 5’, 3’ or intron polymorphisms) were identified (S6 Table). A total of 184, 178, 802 and 603 SNPs were predicted as non-synonymous, 5´, 3´ or intron, and synonymous polymorphisms for gene transcripts (S4 Fig and S6 and S7 Tables). The non-synonymous polymorphisms were used to test hypotheses about their role in resistance (see below).

### Proteomic analysis

Microsomes were assayed for total cytochrome P450 and *b*_5_ content and the highest levels of both were found in the SP strain. Total cytochrome P450 levels in SP, CKR and ROCK were 0.148 (±0.029), 0.075 (±0.058) and 0.070 (±0.008) nmol/mg protein, respectively. Average levels of cytochrome *b*_*5*_ in SP, CKR and ROCK were 0.352 (±0.087), 0.235 (±0.056) and 0.271 (±0.038) nmol/mg protein, respectively (values in parentheses represent the standard deviation of the mean).

**Fig 4:**
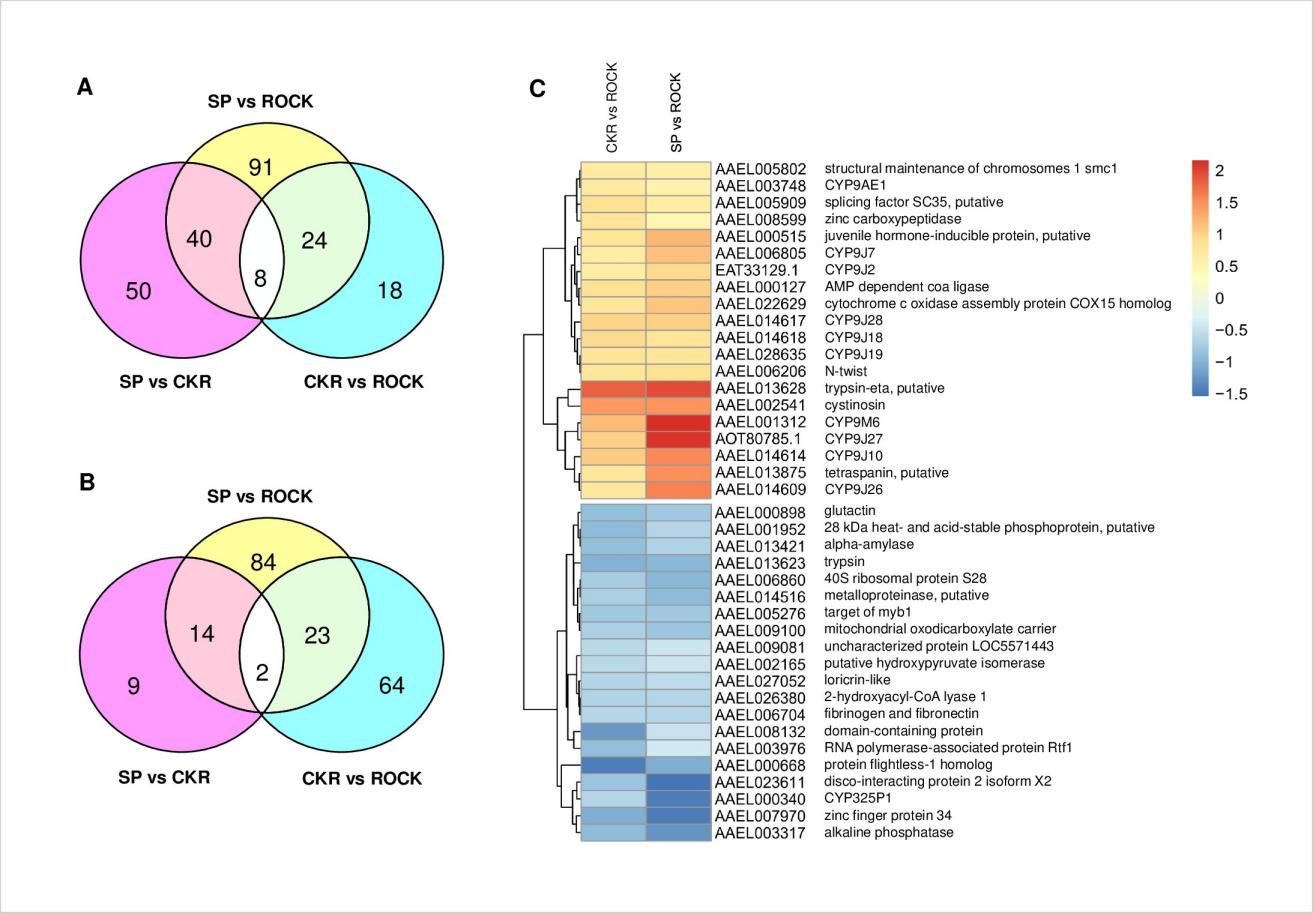
Proteins that were significantly up- or down-regulated between two strains. A: Venn diagram of the numbers of proteins that are significantly up-regulated (p ≤ 0.05) between two strains; B: Venn diagram of the number of genes that are down-regulated (p ≤ 0.05) between two strains; C: Heat map of the top 20 most differentially expressed proteins (based on CKR vs. ROCK comparison, but all genes shown were also differentially expressed in SP relative to ROCK). Heat maps were generated using PHEATMAP (https://www.rdocumentation.org/packages/pheatmap/versions/1.0.12/topics/pheatmap).

We identified 4,487 proteins expressed in microsomes of *A*. *aegypti* and quantified relative expression between strains for 4,003 of those proteins. Volcano plots for the three pairwise comparisons between strains are shown in S5 Fig and the numbers of proteins that were significantly up- or down-regulated (p ≤ 0.05) between two strains are shown in Fig 4 and S8 Table. Relative to the susceptible ROCK strain, a total of 163 and 50 proteins were significantly up-regulated in SP and CKR strains, respectively (Fig 4A), and 32 proteins were up-regulated in both SP and CKR, relative to ROCK (Fig 4A and 4C and S9 Table). This included 16 CYPs (Table 2) (nine *CYP9*s were located in a cluster on chromosome 3), one transcription factor (N-twist, AAEL006206), one putative splicing factor SC35 (AAEL005909) which plays a role in transcript elongation and regulation of alternative splicing [75,76] and cytochrome c oxidase assembly protein COX15 homolog (AAEL022629) which is involved in heme biosynthesis [77].

**Table 2:** Summary of CYPs that were overexpressed in transcriptomic and/or proteomic analyses.

VectorBase accession No.	CYP	mRNA SP/ROCK	Protein SP/ROCK	mRNA CKR/ROCK	Protein CKR/ROCK
AAEL026582	CYP6AA6	1.74	1.03	1.20	0.60
AAEL014893	CYP6BB2	2.94	2.19	1.63	0.55
AAEL003748	CYP9AE1	2.12	0.57	2.28	0.70
AAEL014614	CYP9J10	2.38	1.55	2.04	1.05
AAEL006784	CYP9J17	2.09	0.98	1.38	0.55
AAEL014609	CYP9J26	3.13	1.59	2.23	0.74
AAEL026665	CYP9J27	3.14	2.12	2.37	1.03
AAEL014617	CYP9J28	3.94	1.05	4.25	0.99
AAEL025530	CYP9M5	4.48	1.39	2.89	0.63
AAEL001312	CYP9M6	3.32	2.16	2.14	1.18
AAEL006805	CYP9J2	1.44	1.16		0.66
AAEL009018	CYP6CB1		1.23		0.54
AAEL014618	CYP9J18		0.82		0.96
AAEL028635	CYP9J19		0.79		0.82
AAEL014619	CYP9J22		0.60		0.54
EAT33129.1*	CYP9J7		0.94		0.67

Values indicate the log_2_(FC) of the significantly up-regulated CYPs. Blanks indicate no significant change.

*Protein was not annotated as a gene in AaeL5.1, so NCBI accession number is given.

A total of 123 and 89 proteins were found significantly down-regulated (p ≤ 0.05) in SP and CKR relative to ROCK, respectively (Fig 4B). Among those down-regulated proteins, 25 proteins were down-regulated in both SP and CKR relative to ROCK (Fig 4B and 4C and S9 Table). This included one CYP (CYP325P1) and one putative transcription regulator (zinc finger protein, AAEL007970).

### Comparison of the transcriptomic and proteomic analyses

Among the 4,487 proteins that were detected, 92 were not detected as transcripts (S10 Table). Three of these proteins were differentially expressed in both resistant strains relative to ROCK. Cytochrome c oxidase assembly protein COX15 homolog (AAEL022629) and CYP9J7 (EAT33129.1, unannotated in the genome) were upregulated and uncharacterized protein LOC5571443 (AAEL009081) was down-regulated in SP and CKR strains relative to ROCK. Among the 14,885 genes detected in the transcriptomic data from abdomens, 10,491 were not detected as proteins in the microsomes (S11 Table). In total, >3,850 genes were detected in both the transcriptomic and proteomic data (S12 and S13 Tables). Of these, 54 and 21 genes were differentially expressed in both the transcriptomic and proteomic analyses of SP and CKR, respectively (relative to ROCK) (Tables 3, 4, S14 and S15).

**Table 3:** Summary of non-CYP genes that were up- or down- regulated at both transcript and protein levels in the SP and CKR relative to ROCK.

VectorBase accession No.	Gene name	Chr.	mRNA SP/ROCK	Protein SP/ROCK	mRNA CKR/ROCK	Protein CKR/ROCK
AAEL022232	sulfatase-modifying factor 1 isoform X2	1	3.06	0.53	2.53	0.54
AAEL005412	annexin x	1	3.03	0.53	2.50	0.55
AAEL013421	alpha-amylase	1	-2.05	-0.67	-1.95	-0.89
AAEL002165	putative hydroxypyruvate isomerase	1	-2.38	-0.44	-2.09	-0.59
AAEL026746	fatty acyl-CoA reductase wat-like	3	-2.12	-0.61	-1.50	-0.50
AAEL003317	alkaline phosphatase	3	-2.27	-1.27	-1.78	-0.92
AAEL006704	fibrinogen and fibronectin	3	-1.05	-0.64	-1.51	-0.62
AAEL026380	2-hydroxyacyl-CoA lyase 1	NIGP01001216	-1.54	-0.63	-1.23	-0.68

Values indicate the log_2_(FC) of the up-or down-regulated non-CYP genes. The expression of CYPs is presented in Table 2.

**Table 4:** Comparison of differences found in the RNA-seq and proteomics studies using an unrelated resistant strain, a congenic resistant strain, or both.

		SP	CKR	Fold increase in resolution using a congenic strain (CKR vs. SP)	Both SP and CKR	Fold increase in resolution using multiple strains (SP+CKR vs. SP alone)
Data	Metric	Number			Number	
RNA-seq	Differential expression	1095	456	2.4	366	3.0
RNA-seq	Overexpressed CYPs	22	15	1.5	14	1.6
RNA-seq	Homozygous SNPs^a^	60,527	34,665	1.7	14,350	4.2
Proteomics	Differential expression	286	139	2.1	57	5.0
Proteomics	Overexpressed CYPs	28	16	1.8	16	1.8

^a^See Fig 2.

**Fig 5:**
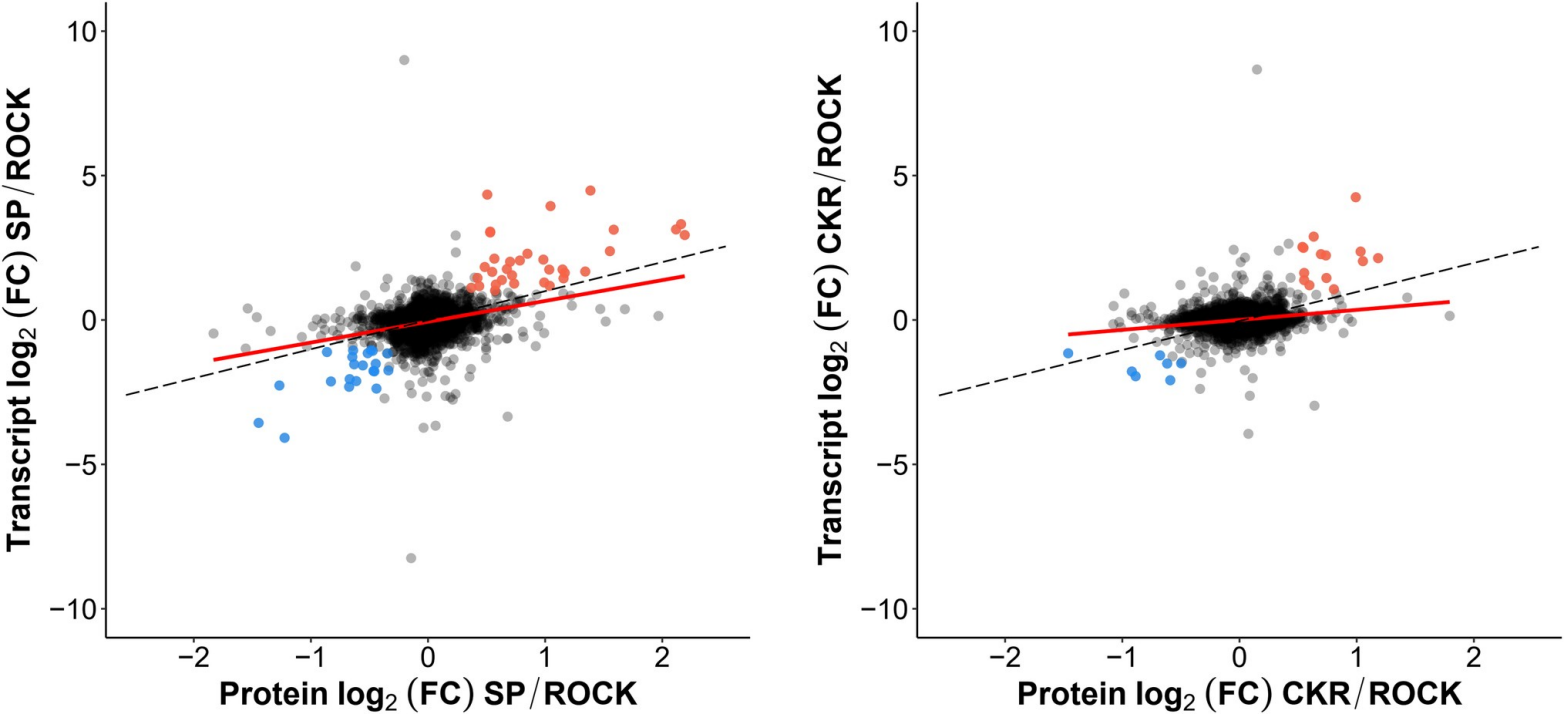
Correlation between all detected proteins and transcripts. A: Plot of the log_2_ (FC) between all detected proteins and transcripts (red line) for SP relative to ROCK (Pearson’s correlation of 0.32). B: Plot of the log_2_ (FC) between all detected proteins and transcripts for CKR relative to ROCK (Pearson’s correlation of 0.20). The dashed line represents a slope of 1.0.

**Fig 6:**
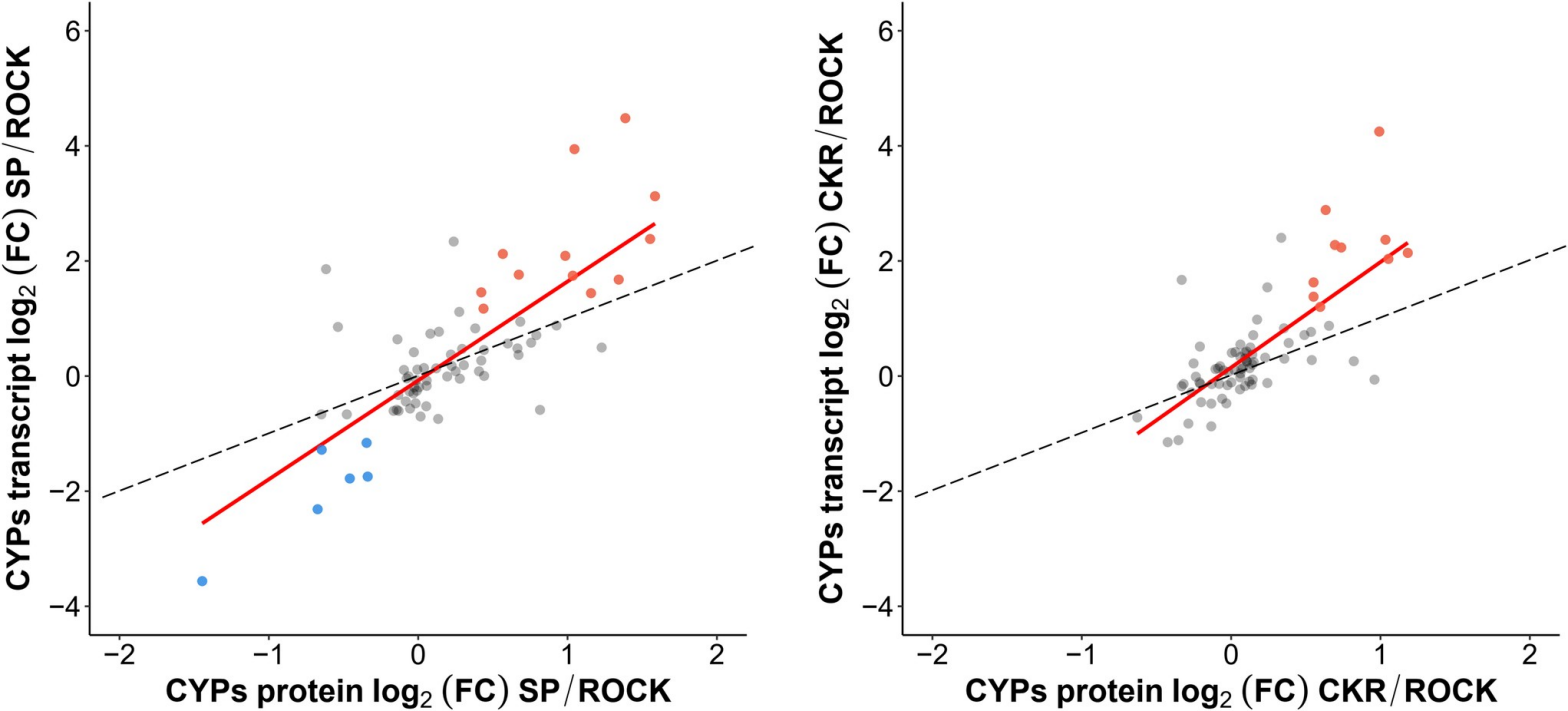
Correlation plots of the log_2_ (FC) between detected CYP proteins and transcripts (red line) for SP and CKR relative to ROCK. A: Correlation plot of the log_2_ (FC) between detected CYP proteins and transcripts for SP relative to ROCK. B: Correlation plot of the log_2_ (FC) between detected CYP proteins and transcripts for CKR relative to ROCK. The dashed line represents a slope of 1.0.

Using all the genes for which we had both transcriptomic and proteomic information (S12 and S13 Tables), there was no significant correlation observed between mRNA and protein expression levels for SP (n = 3851) or CKR (n = 3853) relative to ROCK (Fig 5A and 5B). However, some correlation was found when looking at CYPs alone. A total of 76 CYPs were detected and quantitated at both mRNA and protein levels (S15 Table), and the correlations between transcript and protein log_2_ (FC) values of SP/ROCK and CKR/ROCK were analyzed for those CYPs (Fig 6A and 6B). The Pearson’s correlations were 0.80 and 0.73 for SP/ROCK and CKR/ROCK, respectively. This is likely an overestimation of the correlation because proteins that were not detected as transcripts and transcripts that were not detected as proteins were excluded. Thus, for CYPs, the mRNA levels found in the abdomen can be considered an approximation of protein levels in microsomes, but cannot be considered highly accurate.

The CYPs for which both transcripts and proteins were up- or down-regulated (transcripts and proteins in the same direction) in the SP and CKR strains relative to the ROCK strain are summarized in Table 2 (see also S16 and S17 Tables). Ten CYPs were overexpressed in both the SP and CKR strains as transcripts and proteins, while an additional six CYPs were overexpressed only as proteins (Table 2). All of the overexpressed CYPs belong to the CYP6 and CYP9 families and are the most likely to be responsible for the CYP-mediated resistance. There were no CYPs that were found to be down-regulated in both SP and CKR for both transcript and protein. The non-CYPs for which both transcripts and proteins were up- or down-regulated (transcripts and proteins in the same direction) in the SP and CKR strains relative to the ROCK strain are summarized in Table 3 and none of these genes fits our expectation for being a detoxification enzyme or a regulator of transcription.

Comparison of the transcriptomic and proteomic data suggests that protein stabilization is a mechanism for overexpression of some CYPs in the resistant strains. The CYPs that were found differentially expressed in both resistant strains (as proteins only, or as both proteins and transcripts) are listed in Table 2. Overexpression of both transcript and protein was observed for ten CYPs, consistent with increased transcription as the mechanism of overexpression of these CYPs. In contrast, overexpression of five CYPs (CYP6CB1, CYP9J2, CYP9J18, CYP9J19 and CYP9J22) was observed for protein only. This represents the first data supporting increased protein stabilization as a mechanism of resistance, although more research is needed to test this hypothesis.

### Utility of multiple and/or congenic strains in “omic” analyses of resistance

Studies on the molecular basis of resistance that employ unrelated strains make resolution of the causes of resistance problematic, because too many differences are found and there is no way to distinguish those that are relevant vs. irrelevant to resistance. One approach to improve the resolution of molecular studies is to compare congenic strains. Another approach is to compare multiple strains, assuming the same molecular basis of resistance in all the resistant strains. Herein, we used both approaches and this allows for a comparison of what was gained. As expected, use of a congenic strain resulted in a 1.7- to 2.4-fold increase in resolution (i.e. all genes or proteins identified) relative to using the SP strain alone (Table 4), but a greater improvement in resolution (3.0- to 5.0-fold) was obtained by using both the CKR and SP strains. These gains in resolution were more modest when only CYPs were considered (Table 4). These results indicate that congenic strains improve resolution in RNA-seq and proteomic experiments, but that a greater resolution was found using both SP and CKR.

### Evaluation of hypotheses

Each of the six hypotheses (Table 1) we considered in this study entailed criteria for identifying candidate causes of CYP-mediated resistance. These hypotheses are not necessarily mutually exclusive, and each of them yielded potential causes that fit their specific predictions. For hypothesis #1 (CYP with increased detoxification rate) we searched for CYPs having non-synonymous polymorphisms in both CKR and SP. Two CYPs match these criteria: *CYP325T2* and *CYP9J24* (Table 5). For hypothesis #2 (increased expression of a CYP due to mutation in the promoter of the CYP) we searched for CYPs that were overexpressed (both transcript and protein) in both CKR and SP, relative to ROCK, and ten CYPs matched this criterion (Table 5). For hypothesis #3 (increased expression of one CYP, or one group of tightly linked CYPs, due to duplication) we first searched for CYPs that were overexpressed (both transcript and protein) in both CKR and SP, relative to ROCK and 10 CYPs matched this expectation (Table 5). It is conceivable that a duplication event might involve multiple CYPs, particularly if they are tightly clustered. There were three groups of clustered CYPs that fit this pattern. The first group consists of three neighboring CYPs: *9J26*, *9J27* and *9J28*. The second group consists of *CYP9M5* and *9M6*. The third contains *CYP6AA6* and *6BB2*. For hypothesis #4 we searched for any transcription factors and lncRNAs containing non-synonymous polymorphisms. There were 23 transcription factors matching these criteria (Table 5). For hypothesis #5 we searched for transcription factors and lncRNAs that had altered levels of expression (because we needed to consider both enhancers and repressors) in both resistant strains relative to ROCK. There were 101 that fit this criteria (Table 5). For hypothesis #6 we searched for CYPs that were overexpressed as proteins, but not as transcripts and five CYPs fit this pattern. Collectively, our analyses of the genes fitting the potential patterns for hypotheses #1–6 gave a list of 139 candidate genes. To narrow this list further we refined the above lists to only those genes located at sites associated with homozygous SNPs in the resistant strain across chromosomes 1 and 3 (Fig 3). The genes remaining on this list are shown in Table 5 and are our top candidates for future studies. A gene duplication would be invisible in the chromosome scans (Fig 3), so we would include all ten CYPs including the three CYP clusters (*9J26*-*9J28*, *CYP9M5*-*9M6* and *CYP6AA6*-*6BB2*) as candidates for future studies as well.

**Table 5:** List of factors potentially responsible for CYP-mediated resistance in SP identified by RNA-seq and proteomic analyses. The hypotheses are explained in Table 1. Genes were put into the final column if their position in the genome was consistent with the mapped resistance loci (40Mb-250Mb and 300Mb-310Mb in chromosome 1; 310Mb-320Mb and 330Mb-360Mb in chromosome 3, Fig 3). The VectorBase accession numbers for the CYPs are shown in S18 Table. RednumbersindicatelncRNAs.

Hypothesis^*a*^	Candidates	Candidates after filtering
1	*CYP325T2*, *CYP9J24*	None
2	*CYP6AA6*, *6BB2*, *9AE1*, *9J10*, *9J17*, *9J26-28*, *9M5*, *9M6*	*CYP9AE1*
3	*CYP6AA6*, *6BB2*, *9AE1*, *9J10*, *9J17*, *9J26-28*, *9M5*, *9M6*	*CYP6AA6, 6BB2*, *9AE1*, *9J10*, *9J17*, *9J26-28*, *9M5, 9M6*
4	*AAEL005602*, *006032*, *006035*, *006597*, *007988*, *010576*, *013562*, *013760*, *018695*, *019560*, *019840*, *021303*, *021877*, *022135*, *022195*, *023574*, 023842*, 025336*, *026672*, *026933*, *028025*, *028112*, *028157*	*AAEL005602*, *006032*, *006035*, *006597*, *010576*, *013760*, *018695*, *019560*, *019840*, *021303*, *021877*, *022195*, *023574*, 023842*, 025336*, *026672*, *026933*, *028025*, *028112*
5	*AAEL001459*, *002896*, *003176*, *003506*, *004130*, *004669*, *005247*, *005602*, *005606*, *006197*, 006206, *006932*, 007970, *008401*, *011160*, *012628*, *012990*, *014406*, *019765*, *019809*, *019968*, 020179*, 020308, 020350, 020445, *020459*, 020507, 020816, 020844, 021107, 021117, 021124, 021188, 021207, *021235*, *021303*, 021326, 021614, 021658, 021758*, 021796, 021901, 021935, *021952*, 021984, 022024, 022110, *022402*, 022278*, 022636, 022706, 022711, 023156, 023170, 023172, *023265*, 023302, 023306, 023405, 023428, 023707, 023795*, 023937, 024060, 024161, 024318, 024523*, 024603, 024640, 024782, 024864, 024869, 024872, 024949, 024959, 025193, 025264, 025399, 025559, 025618, 025640*, 025853, 025854, 025912, 026107, 026208, 026242, 026527, *026574*, 026769, 026784, 026801, *026917*, 027182, 027227, 027357, 027461, 027939, 027946, 028219, 028241	*AAEL001459*, *002896*, *003176*, *003506*, *005247*, *005602*, *005606*, *006197*, *008401*, *014406*, *019809*, *019968*, 020179*, 020350, *020459*, 020816, 021117, *021235*, *021303*, 021614, 021658, 021758*, 021796, 021901, *021952*, 022278*, 022706, 022711, 023156, *023265*, 023306, 023795*, 024523*, 024603, 024640, 024782, 024864, 024869, 025618, 025640*, 026107, 026242, 026527, *026574*, 026784, 027227, 027357, 027461
6^	CYP6CB1, CYP9J18, CYP9J19, CYP9J2, CYP9J22	CYP6CB1, CYP9J18, CYP9J19, CYP9J2, CYP9J22

^*a*^The hypotheses are described in Table 1.

*Gene on an unplaced scaffold that could not be filtered.

^Factor causing stabilization of these CYPs could not be mapped (was unknown), so no filtering could be applied.

## Discussion

Understanding the molecular basis underlying CYP-mediated resistance has been a major challenge in the field of insecticide resistance research. Here, we were able to narrow down the list of candidate genes and lncRNAs causing pyrethroid resistance in the CKR strain of *A*. *aegypti* to 80 (Table 5). 65 are transcriptional factors or lncRNAs that could be responsible for the upregulation of one or more CYPs, leading to resistance. From our results, we narrowed down the molecular basis of CYP-mediated resistance in SP to the three most plausible hypotheses: That the overexpression of *CYPs* is due to non-synonymous mutations in a switch (transcription factor or lncRNA), due to mutations in the promoter of a switch (changing the amount of switch transcribed) and/or due to stabilization of CYP proteins. The possible link between the overexpression of the *TRAP subunit alpha-like* gene and stabilization of CYP proteins requires further investigation.

One or more CYPs are overexpressed and ultimately responsible for part of the pyrethroid resistance in the CKR strain (independent of the mechanism by which the CYPs(s) are overexpressed). For decades, studies of insecticide resistance have measured CYP transcripts (from whole bodies or from body regions) as a surrogate for measuring CYP protein levels in microsomes. Our results indicate this is not an ideal approach because the correlation between CYP transcript and protein levels is not high and transcript measurements may miss the presence of important CYPs that are only detected as proteins. For example, the proteomics study identified six CYPs that were up-regulated (p ≤ 0.05) in SP and CKR relative to ROCK that were not found using RNA-seq (one of these because the gene was not properly annotated in the genome). Thus, use of CYP transcript abundance, as a surrogate for measuring protein levels is not wrong, but can be fallible.

CYP-mediated resistance is commonly associated with increased levels of total cytochromes P450 and in some cases cytochrome *b*_*5*_ and/or P450 reductase [62], although in one study only the elevated levels of cytochrome *b*_*5*_ were genetically linked to resistance [78]. Cytochrome b_5_ was previously found to be overexpressed in the SP strain [36] and we confirm that result (spectrophotometrically, and with both RNA-seq and proteomics). However, the CKR strain did not have elevated levels of P450 reductase or *b*_*5*_, suggesting that overexpression of these proteins is not needed for CYP-mediated resistance in CKR.

The initial work on the SP strain used microarrays and found 15 *CYP*s up-regulated >3-fold in females relative to the susceptible SMK strain [36]. Our RNA-seq data confirms the overexpression of *CYP6BB2*, *9M5* and *9M6* found in the microarray data, but overall the list of overexpressed CYPs was considerably different between these approaches. These differences appear to be driven primarily by the source of the RNA (3 d old whole females for the microarray and 4–7 d old adult female abdomens for the RNA-seq), rather than the differences in the susceptible strains used, because a study using RT-qPCR with 5–7 d old females (whole bodies) confirmed that *CYP6F2*, *6F3*, *6Z7*, *9M5* and *9M6* were overexpressed in both the SP and CKR strains relative to the susceptible ROCK strain [14].

The SNP analysis using RNA-seq data with SP and two congenic strains successfully identified the resistance locus on chromosome 3 (containing *Vssc*), suggested there may be a second resistance locus on chromosome 3, and also indicated there were multiple resistance loci on chromosome 1 (Fig 3). There were no resistance loci found on chromosome 2. In a previous quantitative trait loci (QTL) mapping study of permethrin resistance in *A*. *aegypti*, besides a SNP found in *Vssc*, a QTL affecting survival was also found at the 3´ end of chromosome 1 [79]. Overall, our study provided a reasonable resistance locus mapping method with the combined techniques of congenic strains and transcriptomics, although use of DNA sequences for a bulked segregant analysis would likely yield higher resolution.

Comparison of the genes we identified as differentially expressed in our resistant strains to previous studies reveals large differences. Previous transcriptomic analyses of pyrethroid resistance in other strains of *A*. *aegypti* identified hundreds of transcripts upregulated in the resistant strains [80,81]. Consistent with what was found in one of those studies [80], we identified *AAEL014406* as underexpressed in our resistant strains (Table 5, genes passing the filters). None of the other genes we identified (Table 5, genes passing the filters) were identified in the previous transcriptomic analyses. Similarly, searches of the ten most highly expressed genes from these previous studies with our data found that none of these previously identified genes were overexpressed in our resistant strains. Candidate genes previously associated with pyrethroid resistance in other species of mosquitoes include transferrin [82], iron responsive element binding protein [83], glycogen branching enzyme [84], chymotrypsin [82] and G-protein coupled receptor [85]. None of these genes were overexpressed in our resistant strains, leading us to conclude that they do not play a role in pyrethroid resistance in CKR or SP.

Our results confirmed that CYP-mediated resistance in SP and CKR is associated with the increased expression level of multiple CYPs (both transcript and protein levels), including CYP6AA6, 6BB2, 9AE1, 9M5, 9M6, 9J10, 9J17, 9J26, 9J27 and 9J28, and we identified a list of candidate genes and lncRNAs for future investigation. These include mutations in CYPs, overexpression of groups of CYPs (potentially due to gene amplification), transcription factors plus lncRNAs and stabilization of CYP proteins. Our results suggest the presence of multiple resistance loci on chromosome 1 and perhaps two loci on chromosome 3. Future studies to increase the resolution of the resistance loci, and to examine the candidate genes identified here will propel our understanding of CYP-mediated resistance in *A*. *aegypti* forward.

## Supporting information

S1 Table Summary of RNA-seq metrics from the *Aedes aegypti* transcriptomes. (DOCX)

S2 Table Genes and lncRNAs detected in the ROCK, SP and CKR strains. (XLSX)

S3 Table The edgeR output for relative expression of genes in pairwise comparison between two strains. (XLSX)

S4 Table Significantly up-regulated genes and lncRNAs in both the SP and CKR strains relative to the ROCK strain in transcriptomic analysis. Putative transcription regulators are highlighted.
(XLSX)

S5 Table Significantly down-regulated genes and lncRNAs in both the SP and CKR strains relative to the ROCK strain in transcriptomic analysis. Putative transcription regulators are highlighted.
(XLSX)

S6 Table Homozygous single nucleotide polymorphisms (SNPs) found in both the SP and CKR strains, but different from the ROCK and LVP strains. (CSV)

S7 Table Homozygous non-synonymous polymorphisms found in both the SP and CKR strains, but different from the ROCK and LVP strains. Putative transcription regulators are highlighted. CYPs are shown in red.
(XLSX)

S8 Table Significantly up- or down-regulated proteins in pairwise comparisons between strains. (XLSX)

S9 Table Significantly up- or down-regulated proteins in both the SP and CKR strains relative to the ROCK strain in proteomic analysis. (XLSX)

S10 Table Proteins detected in proteomic analysis, but not in transcriptomic analysis. Significantly up- or down- regulated proteins in the SP and CKR strains relative to the ROCK strain were highlighted in red and green, respectively.
(XLSX)

S11 Table Genes and lncRNAs detected in transcriptomic analysis, but not in proteomic analysis. (XLSX)

S12 Table Genes with quantitative data of expression level in the SP strain relative to ROCK in both the transcriptomic and proteomic analyses. The CYPs are named for convenience.
(XLSX)

S13 Table Genes with quantitative data of expression level in the CKR strain relative to ROCK in both the transcriptomic and proteomic analyses. The CYPs are named for convenience.
(XLSX)

S14 Table Significantly up- or down-regulated genes in both mRNA and protein levels in the SP strain relative to ROCK. (XLSX)

S15 Table Significantly up- or down-regulated genes in both mRNA and protein levels in the CKR strain relative to ROCK. (XLSX)

S16 Table CYPs having quantitative data for transcripts and proteins in the SP and CKR strains relative to ROCK. (XLSX)

S17 Table Significantly up- or down-regulated CYPs in both mRNA and protein levels in the SP or CKR strain relative to ROCK. (XLSX)

S18 Table *CYP* accession numbers in AaeL5.1. (XLSX)

S1 Fig The approaches taken to evaluate patterns of expression and polymorphisms relative to hypothesis about the cause(s) of resistance. Arrows indicate the flow of information or samples.
(TIF)

S2 Fig Multidimensional scaling (MDS) plot showing relationships between different samples. Distance between each label indicates similarity.
(TIF)

S3 Fig Volcano plots for transcriptomic analysis. **A: Volcano plot for SP/ROCK; B: Volcano plot for CKR/ROCK; C: Volcano plot for SP/CKR.** The significantly up- (red dots) or down-regulated (blue dots) (log_2_ (FC) ≥ 1 or log_2_ (FC) ≤ -1, and FDR ≤ 0.01) genes and lncRNAs.
(TIF)

S4 Fig Venn diagrams of different types of homozygous SNPs in ROCK, SP and CKR strains under the condition SP≠LVP. A: Venn diagram of non-synonymous polymorphisms; B: Venn diagram of 5’ polymorphisms; C: Venn diagram of 3’ and intron polymorphisms. a: stands for the number of homozygous SNPs met the criteria ROCK ≠ (CKR = SP); b: stands for the number of homozygous SNPs meeting the criteria (ROCK = CKR) ≠ SP; c: stands for the number of homozygous SNPs meeting the criteria (ROCK = SP) ≠ CKR; d: stands for the number of homozygous SNPs meeting the criteria ROCK = SP = CKR.
(TIF)

S5 Fig Volcano plots from proteomic analysis. A: Volcano plot for SP/ROCK; B: Volcano plot for CKR/ROCK; C: Volcano plot for SP/CKR. The significantly up- (red dots) or down-regulated (blue dots) (p ≤ 0.05) genes and lncRNAs.
(TIF)
