# Supplementary material for: Transcriptomic and proteomic analysis of pyrethroid resistance in the CKR strain of Aedes aegypti
Source: PLoS Negl Trop Dis. 2021 Nov 1;15(11):e0009871. doi: 10.1371/journal.pntd.0009871 (PMC8559961; doi:10.1371/journal.pntd.0009871)
Supplement: S1 Table — (DOCX) [file pntd.0009871.s001.docx]

S1 Table. Summary of RNA-seq metrics from the *Aedes aegypti* transcriptomes.

| Library | Total reads | Mapped reads^1^ | Ambiguous mapped reads^2^ (%) | Overall mapping rate (%) |
| --- | --- | --- | --- | --- |
| ROCK1 | 24,303,071 | 21,794,910 | 1,650,098 (7.6%) | 89.68 |
| ROCK2 | 35,602,043 | 32,126,372 | 2,511,083 (7.8%) | 90.24 |
| ROCK3 | 26,083,076 | 23,391,004 | 1,787,251 (7.6%) | 89.68 |
| ROCK4 | 40,978,915 | 37,026,051 | 2,778,490 (7.5%) | 90.35 |
| SP1 | 37,972,993 | 33,996,661 | 2,627,765 (7.7%) | 89.53 |
| SP2 | 41,556,225 | 37,179,937 | 2,916,059 (7.8%) | 89.47 |
| SP3 | 33,699,587 | 30,132,599 | 2,346,274 (7.8%) | 89.42 |
| SP4 | 37,550,854 | 33,551,916 | 2,569,636 (7.7%) | 89.35 |
| CKR1 | 36,445,811 | 32,626,403 | 2,418,185 (7.4%) | 89.52 |
| CKR2 | 39,431,617 | 35,372,655 | 2,569,577 (7.3%) | 89.71 |
| CKR3 | 37,639,793 | 33,840,992 | 2,487,852 (7.4%) | 89.91 |
| CKR4 | 37,514,549 | 33,782,274 | 2,663,071 (7.9%) | 90.05 |

^1^ Overall reads that mapped to the reference genome database AaegL5.1.

^2^ Among mapped reads, reads that have multiple alignments.
