# Supplementary figures and images for: Transcriptomic and proteomic analysis of pyrethroid resistance in the CKR strain of Aedes aegypti
Source: PLoS Negl Trop Dis. 2021 Nov 1;15(11):e0009871. doi: 10.1371/journal.pntd.0009871 (PMC8559961; doi:10.1371/journal.pntd.0009871)

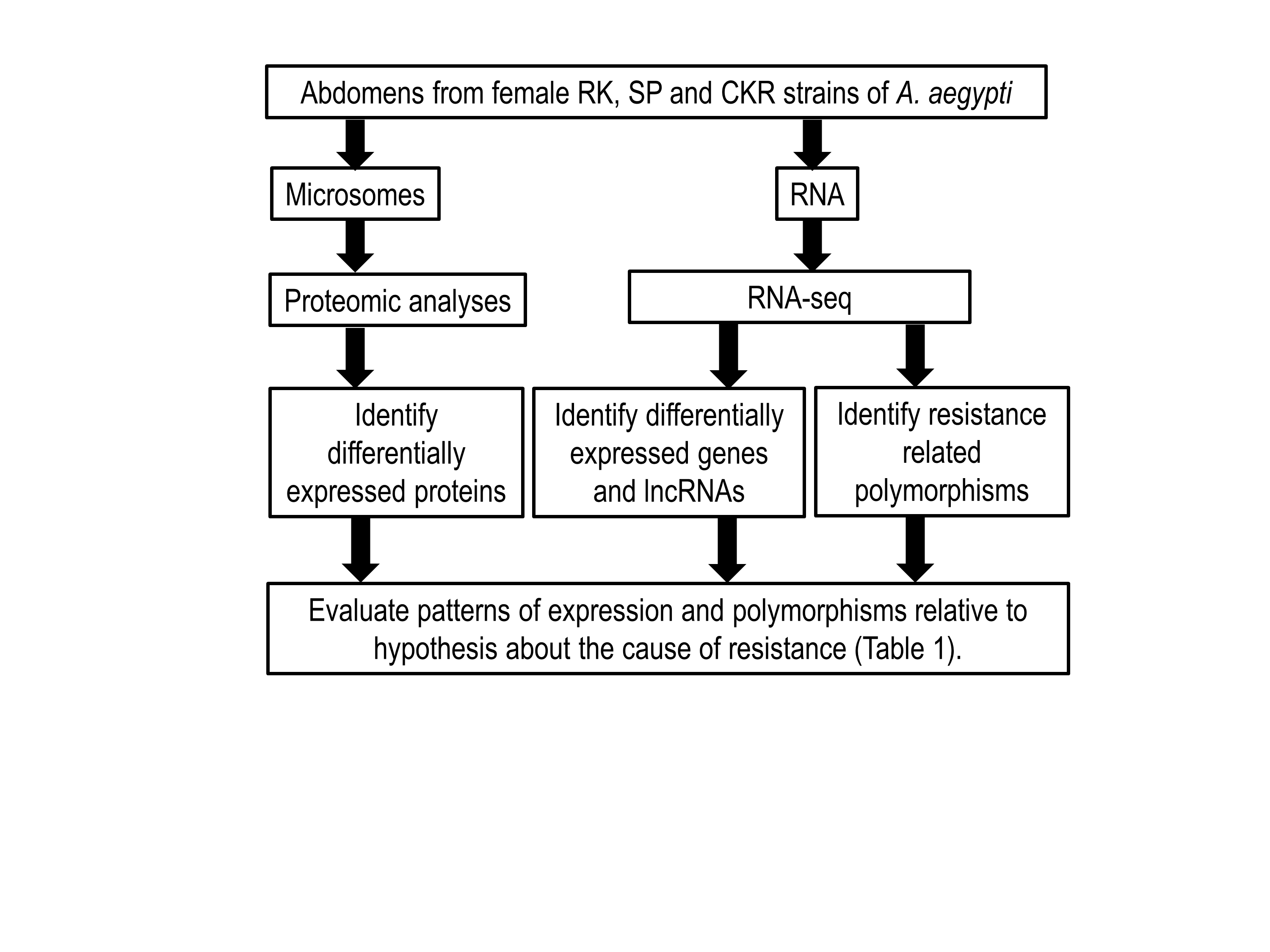

Supplement: S1 Fig — Arrows indicate the flow of information or samples. (TIF) [file pntd.0009871.s019.tif]

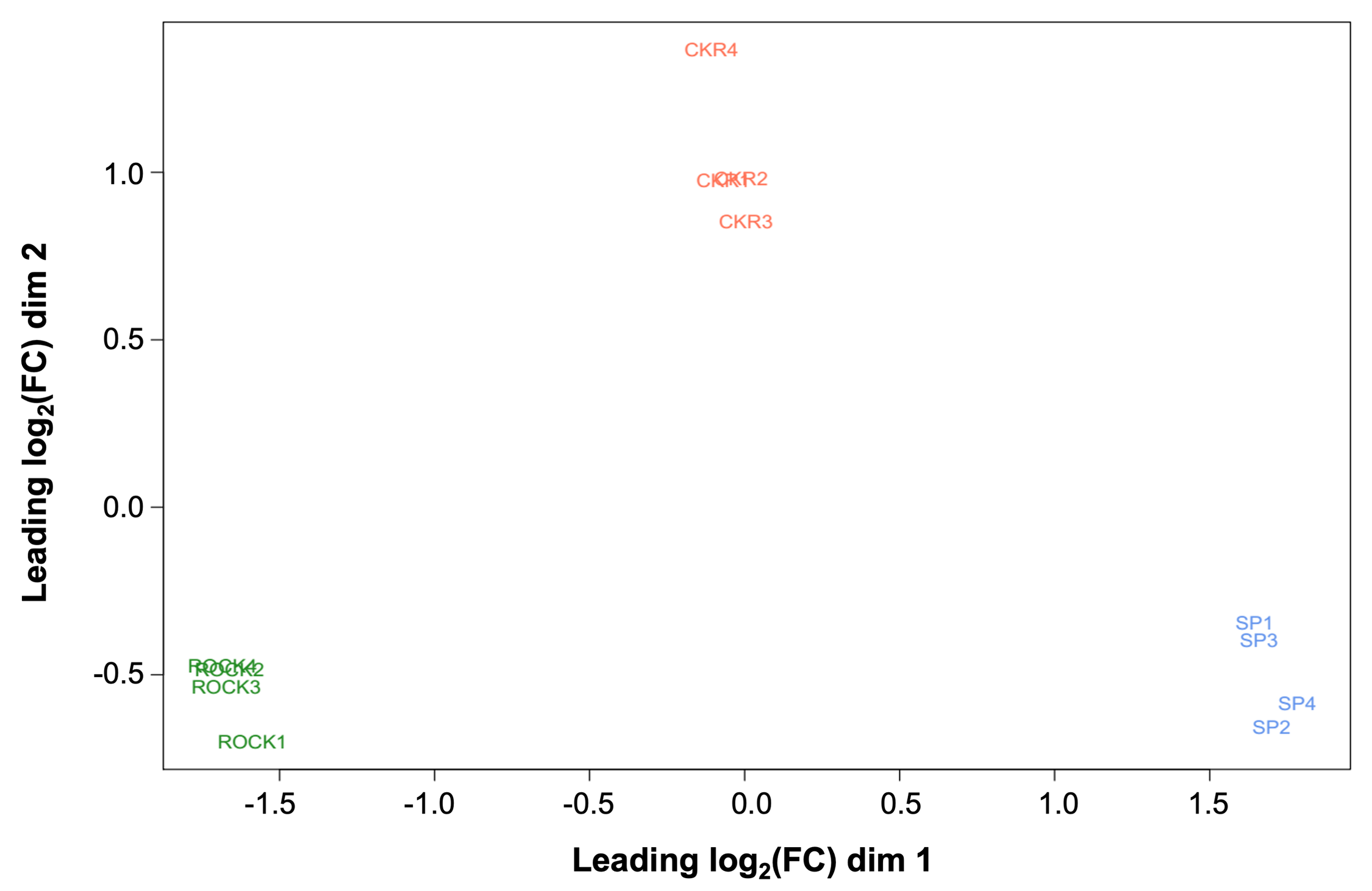

Supplement: S2 Fig — Distance between each label indicates similarity. (TIF) [file pntd.0009871.s020.tif]

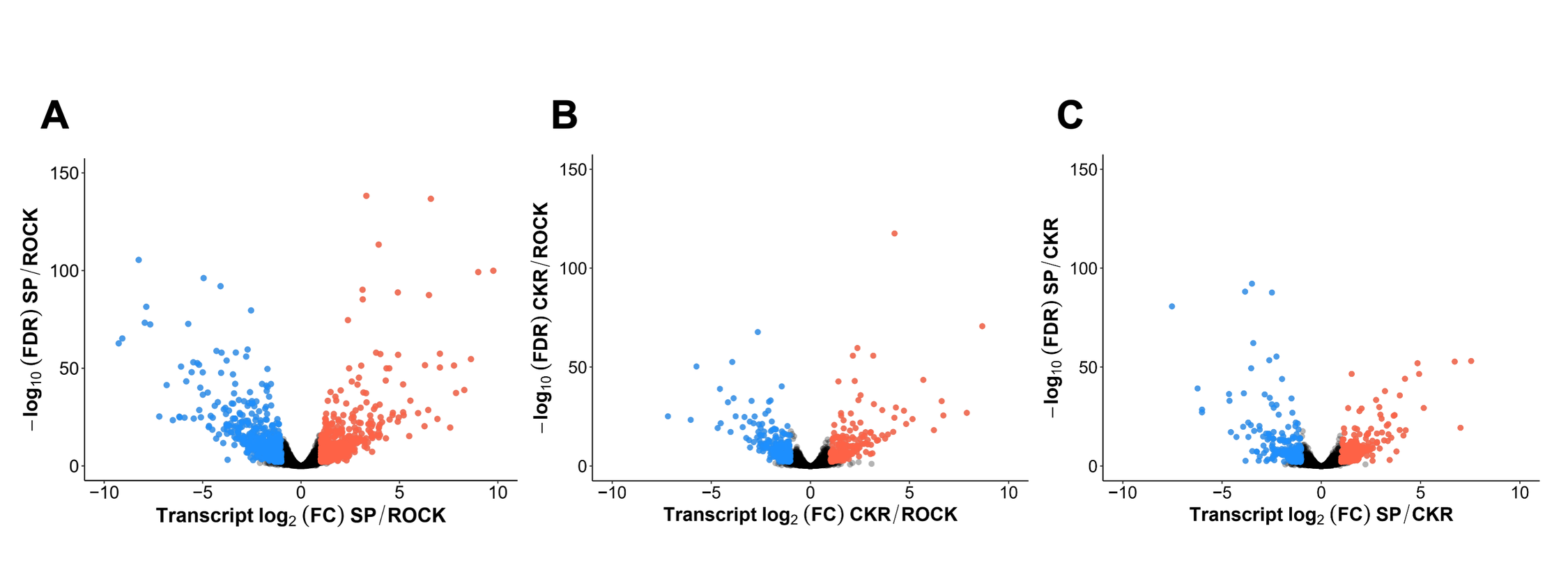

Supplement: S3 Fig — A: Volcano plot for SP/ROCK; B: Volcano plot for CKR/ROCK; C: Volcano plot for SP/CKR. The significantly up- (red dots) or down-regulated (blue dots) (log2 (FC) ≥ 1 or log2 (FC) ≤ -1, and FDR ≤ 0.01) genes and lncRNAs. (TIF) [file pntd.0009871.s021.tif]

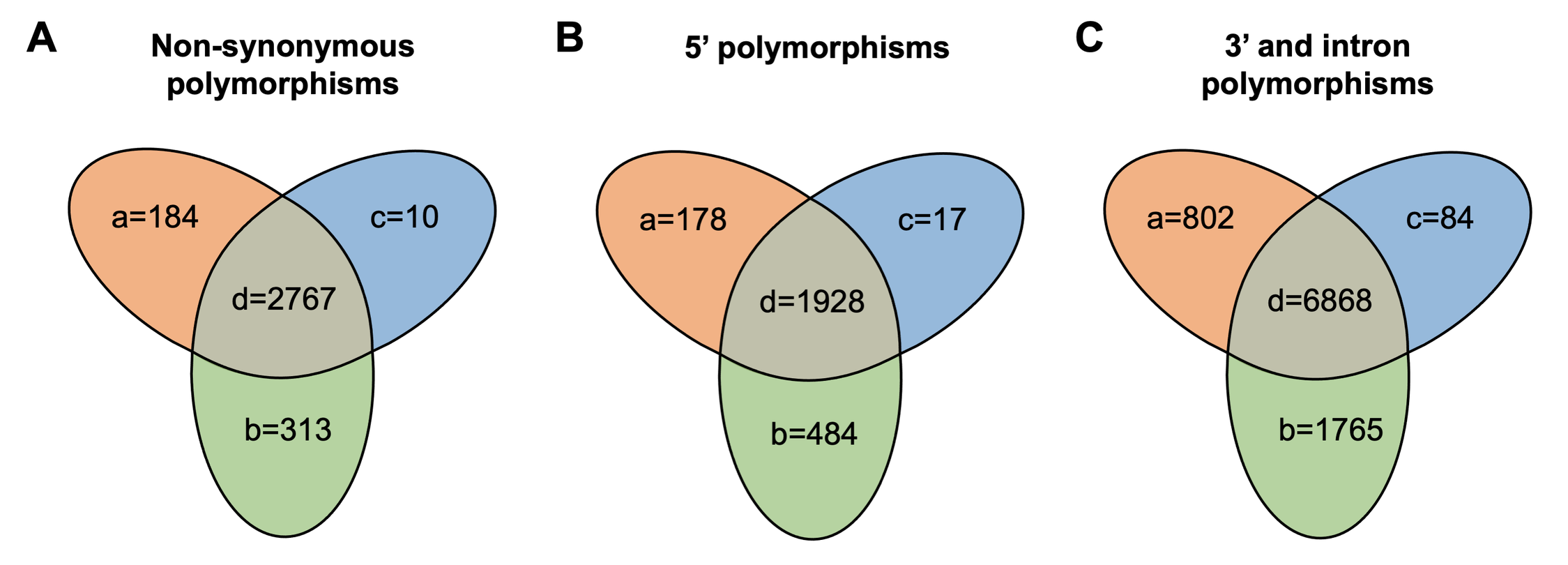

Supplement: S4 Fig — A: Venn diagram of non-synonymous polymorphisms; B: Venn diagram of 5’ polymorphisms; C: Venn diagram of 3’ and intron polymorphisms. a: stands for the number of homozygous SNPs met the criteria ROCK ≠ (CKR = SP); b: stands for the number of homozygous SNPs meeting the criteria (ROCK = CKR) ≠ SP; c: stands for the number of homozygous SNPs meeting the criteria (ROCK = SP) ≠ CKR; d: stands for the number of homozygous SNPs meeting the criteria ROCK = SP = CKR. (TIF) [file pntd.0009871.s022.tif]

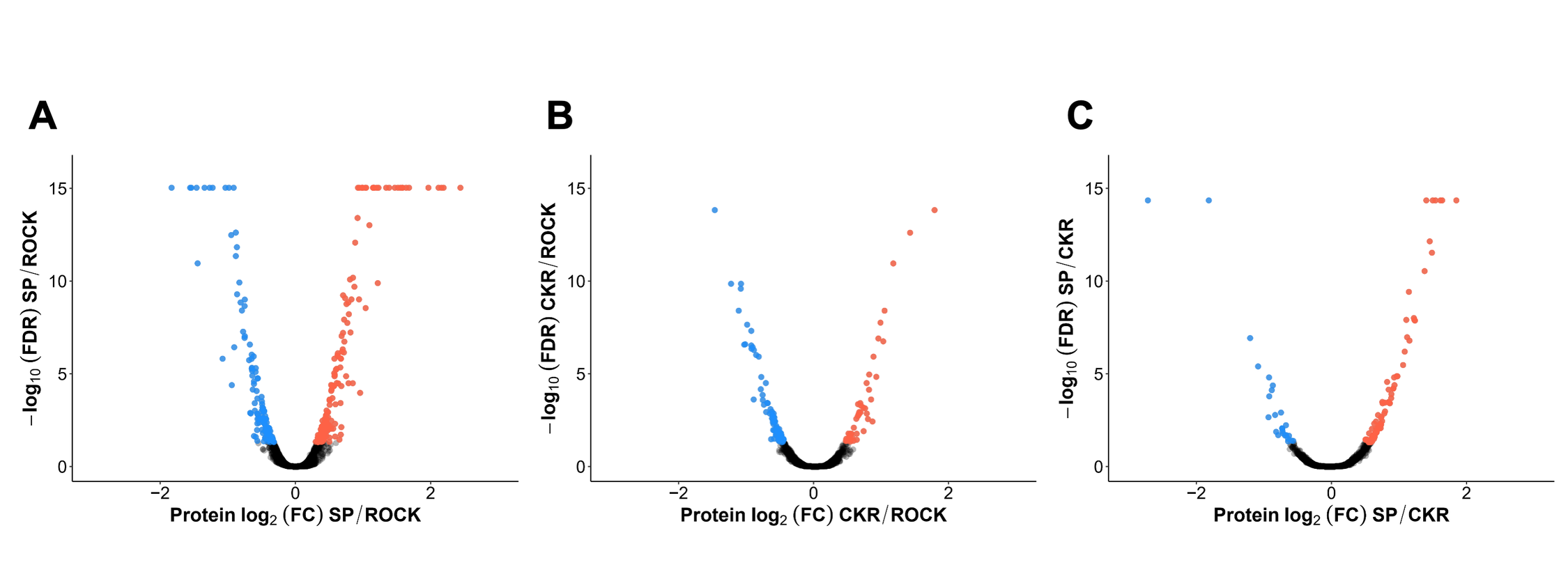

Supplement: S5 Fig — A: Volcano plot for SP/ROCK; B: Volcano plot for CKR/ROCK; C: Volcano plot for SP/CKR. The significantly up- (red dots) or down-regulated (blue dots) (p ≤ 0.05) genes and lncRNAs. (TIF) [file pntd.0009871.s023.tif]
